# Development and evaluation of an up-converting phosphor technology-based lateral flow assay for the rapid, simultaneous detection of *Vibrio cholerae* serogroups O1 and O139

**DOI:** 10.1371/journal.pone.0179937

**Published:** 2017-06-29

**Authors:** Min Hao, Pingping Zhang, Baisheng Li, Xiao Liu, Yong Zhao, Hailing Tan, Chongyun Sun, Xiaochen Wang, Xinrui Wang, Haiyan Qiu, Duochun Wang, Baowei Diao, Huaiqi Jing, Ruifu Yang, Biao Kan, Lei Zhou

**Affiliations:** 1 State Key Laboratory for Infectious Disease Prevention and Control, National Institute for Communicable Disease Control and Prevention, Chinese Center for Disease Control and Prevention, Beijing, P. R. China; 2 Beijing Chaoyang District Center for Disease Control and Prevention, Beijing, P. R. China; 3 Laboratory of Analytical Microbiology, State Key Laboratory of Pathogen and Biosecurity, Beijing Institute of Microbiology and Epidemiology, Beijing, P. R. China; 4 Beijing Key Laboratory of POCT for Bioemergency and Clinic (No. BZ0329), Beijing, P. R. China; 5 Guangdong Provincial Center for Disease Control and Prevention, Guangzhou, Guangdong, P. R. China; 6 Chongqing Entry Exit Inspection and Quarantine Bureau, Chongqing, P. R. China; 7 Department of Clinical Laboratory, Chinese People’s Liberation Army General Hospital, Beijing, P. R. China; 8 College of Animal Science and Technology, Jilin Agricultural University, Changchun, Jilin, P. R. China; 9 Institute for Plague Prevention and Control of Hebei Province, Zhangjiakou, Hebei, P. R. China; 10 Collaborative Innovation Center for Diagnosis and Treatment of Infectious Diseases, Hangzhou, Zhejiang, P. R. China; 11 College of Life Sciences, Northwest University, Xi’an, Shanxi, P. R. China; 12 National Engineering Research Center for Miniaturized Detection Systems, Northwest University, Xi’an, Shanxi, P. R. China; Midwestern University, UNITED STATES

## Abstract

*Vibrio cholerae* serogroups O1 and O139 are etiological agents of cholera, a serious and acute diarrheal disease, and rapid detection of *V*. *cholerae* is a key method for preventing and controlling cholera epidemics. Here, a point of care testing (POCT) method called Vch-UPT-LF, which is an up-converting phosphor technology-based lateral flow (UPT-LF) assay with a dual-target detection mode, was developed to detect *V*. *cholerae* O1 and O139 simultaneously from one sample loading. Although applying an independent reaction pair made both detection results for the two Vch-UPT-LF detection channels more stable, the sensitivity slightly declined from 10^4^ to 10^5^ colony-forming units (CFU) mL^−1^ compared with that of the single-target assay, while the quantification ranges covering four orders of magnitude were maintained. The strip showed excellent specificity for seven *Vibrio* species that are highly related genetically, and nine food-borne species whose transmission routes are similar to those of *V*. *cholerae*. The legitimate arrangement of the two adjacent test lines lessened the mutual impact of the quantitation results between the two targets, and the quantification values did not differ by more than one order of magnitude when the samples contained high concentrations of both *V*. *cholerae* O1 and O139. Under pre-incubation conditions, 1×10^1^ CFU mL^−1^ of *V*. *cholerae* O1 or O139 could be detected in fewer than 7 h, while the Vch-UPT-LF assay exhibited sensitivity as high as a real-time fluorescent polymerase chain reaction with fewer false-positive results. Therefore, successful development of Vch-UPT-LF as a dual-target assay for quantitative detection makes this assay a good candidate POCT method for the detection and surveillance of epidemic cholera.

## Introduction

Cholera is a seriously infectious, acute, epidemic diarrheal disease caused by *Vibrio cholerae* worldwide, and especially in Asia, Africa and South America [1,2]. According to the World Health Organization, an estimated 21000 to 143000 deaths are reported for 1.3 to 4.0 million cases of cholera each year [3]. Most epidemics have been caused by *V*. *cholerae* serogroups O1 and O139 among the more than 200 *V*. *cholerae* serogroups [1,2]. *V*. *cholerae* O1 has been shown to be responsible for the sixth and seventh cholera pandemics [2]. *V*. *cholerae* O139 emerged in 1992, and it has caused epidemics in south and east Asia [4]. Plankton is the known natural reservoir for *V*. *cholerae* [5,6], and people often become infected through ingestion of contaminated water [7] or seafood [8], and are even infected through household contacts [9]. Both *V*. *cholerae* O1 and O139 can produce an enterotoxin, referred to as cholera toxin, which causes serious diarrhea, causing dehydration, and can lead to death in a few days in 50% of infected patients in the absence of rehydration therapy [2]. Because rehydration therapy and oral medicine are effective for such patients [2,10], it is important to prevent and control cholera epidemics with surveillance using point of care testing (POCT) methods to find and manage cases of infection on site and in good time [4,11].

Current detection methods for cholera include culture-based, nucleic acid-based, and immunological methods. Compared with traditional culture-based detection methods, nucleic acid-based detection methods have led to increasingly frequent detections of pathogenic microorganisms [12–17], whereas the combination of these two methods provides a powerful tool for the detection, isolation, and characterization of *V*. *cholerae* [18,19]. Immunological methods [20] have become extremely important during the early infection period. For example, *V*. *cholerae* could be identified in environmental samples during an inter-epidemic period in Bangladesh using immunological methods, even though the *V*. *cholerae* in these samples could not be cultured [9]. Currently, although these methods are sufficiently sensitive and accurate, the need for expensive equipment and professional training is a barrier to their use in the field and in primary laboratories. The sensitivity of traditional POCT methods such as the agglutination test [21–23] is too low for reliable application, while an easily quenched fluorescent group makes fluorescent assays unstable [24], although they are sufficiently sensitive. An immune-chromatographic strip test that uses colloidal gold nanoparticles as bio-labels is a common POCT method; however, the results of the test, especially for samples with low concentrations of target analytes or interfering contaminants, are difficult to assess because they are based on observations using the naked eye [25,26]. Therefore, rapid, simple, sensitive, and accurate POCT detection methods are still needed. Recently, an up-converting phosphor technology-based lateral flow (UPT-LF) assay using up-converting phosphor particles (UCPs) as the bio-label has been developed as a new POCT method, and it exhibits high sensitivity and stability, as well as robust performance, when tested with complex samples [27–30], including those containing multiple targets [27,30]. In the current study, a UPT-LF strip for the rapid and simultaneous detection of *V*. *cholerae* serogroups O1 and O139 was established. Differing from multiplex detection apparatus by simple assemblage of many single-target strips in a disc [27,30], this dual-target assay could quantitatively detect two targets in a strip, once, by immobilizing two antibodies specific for each of the two targets in adjacent positions on a strip-format nitrocellulose membrane. In addition, the quality control signals in this dual-target assay can be maintained by relying on the application of an independent reaction pair, which is quite different from that of the single-target strip [28,29], thereby avoiding any mutual interference between the quantitation results for the two targets. The performance of the UPT-LF assay was extensively evaluated. This included an assessment of specificity, simultaneous detection of O1 and O139, determination of detection time under pre-incubation conditions, as well as comparison with a real-time fluorescent polymerase chain reaction (PCR) assay, a colloidal gold assay and a culture method for the analysis of field water samples.

## Materials and methods

### Ethics statement

BALB/c mice (8 weeks old) were used for the production of monoclonal antibodies (mAb), while New Zealand white rabbits were used for polyclonal antibodies. All animal experiments were conducted in compliance with the Guidelines for the Welfare and Ethics of Laboratory Animals of China, in the facility of Laboratory Animal Research Center accredited by the Beijing Institute of Microbiology and Epidemiology (Beijing, China). All experimental protocols about vertebrate works concerning the preparation of monoclonal and polyclonal antibodies were approved by the Committee of the Welfare and Ethics of Laboratory Animals, the Chinese Center for Disease Control and Prevention (Beijing, China), as well as Beijing Institute of Microbiology and Epidemiology (Beijing, China). All experimental manipulations were reviewed as part of obtaining the field permit. After the mice were culled by cervical dislocation humanely, their spleens were dissected for cell fusion to prepare monoclonal antibodies. The sera used to prepare polyclonal antibodies were collected from the carotid arteries of the immunized, deeply anesthetized rabbits using 3% pentobarbital.

Field water samples were obtained from 24 sampling sites in public places; these are all located near the Zhujiang River estuary (Geographic coordinate: 113° East, 23° North) in Guangzhou City, Guangdong Province, China. Permission to sample was issued by the Guangdong Provincial Center for Disease Control and Prevention. No protected species were sampled.

### Reagents and materials

UCPs (NaYF4:Yb^3+^, Er^3+^) with excitation and emission spectrum peaks of 980 nm and 541.5 nm, respectively, and of approximately 50 nm in diameter, were prepared and provided by Dr. Yan Zheng, Shanghai Kerune Phosphor Technology Co., Ltd. (Shanghai, China). Glass fibers (GFCP20300) and nitrocellulose membranes (SHE 1350225) were purchased from Millipore Corp. (Bedford, MA, USA). Absorbent papers (No. 470 and No. 903) were purchased from Schleicher & Schuell, Inc. (Keene, NH, USA). Plastic cartridges were designed by our group and manufactured by Shenzhen Jincanhua Industry Co. (Shenzhen, China). Laminating cards were obtained from Shanghai Liangxin Biotech Co. (Shanghai, China). Tryptone and yeast extract were purchased from Oxiod Co., Ltd. (Hampshire, United Kingdom). Goat anti-mouse IgG, goat IgG, and rabbit anti-goat IgG were saved by the Laboratory of Analytical Microbiology, Beijing Institute of Microbiology and Epidemiology. The UPT biosensor was designed and assembled by our research group and Shanghai Institute of Optics and Fine Mechanics, Chinese Academy of Sciences (Shanghai, China).

*V*. *cholerae* O1 N16961 (international standard strain, the representative strain of the seventh cholera pandemic) and *V*. *cholerae* O139 MO45 (international standard strain, a newly discovered strain from the Indian and Bangladeshi cholera epidemics in 1992) were used to test the assay detection sensitivity. The bacterial strains applied for performance evaluations were listed in Table 1. All bacterial strains were stored by State Key Laboratory for Infectious Disease Prevention and Control, Chinese Center for Disease Control and Prevention (Beijing, China).

**Table 1:** Bacterial strains applied for the performance evaluations of up-converting phosphor technology-based lateral flow assay.

Performance evaluation	Bacterial strain	Amount of isolates
**Sensitivity of VchO1-UPT-LF**^**a**^	*V*. *cholerae* O1 N16961	1
**Sensitivity of VchO139-UPT-LF**^**b**^	*V*. *cholerae* O139 MO45	1
**Specificity of VchO1-UPT-LF**^**a**^	*V*. *cholerae* serogroup O139	15
**Specificity of VchO139-UPT-LF**^**b**^	*V*. *cholerae* serogroup O1	15
**Specificity of VchO1-UPT-LF**^**a**^ **and VchO139-UPT-LF**^**b**^	*V*. *cholerae* serogroup non-O1/non-O139	15
**Specificity of VchO1-UPT-LF**^**a**^**, VchO139-UPT-LF**^**b**^ **and Vch-UPT-LF**^**c**^	*Vibrio fluvialis*	1
	*Vibrio metschnikovii*	1
	*Vibrio mimicus*	1
	*Vibrio vulnificus*	2
	*Vibrio parahaemolyticus*	2
	*Aeromonas hydrophila*	2
	*Escherichia coli*	2
	*Salmonella*	3
	*Shigella flexneri*	2

^a^ the up-converting phosphor technology-based lateral flow assay for the rapid detection of *V*. *cholerae* serogroups O1

^b^ the up-converting phosphor technology-based lateral flow assay for the rapid detection of *V*. *cholerae* serogroups O139

^c^ the up-converting phosphor technology-based lateral flow assay for the rapid and simultaneous detection of *V*. *cholerae* serogroups O1 and O139.

### Bacterial culture and antibody preparation

*Vibrio* spp. strains were cultured in alkaline peptone water (APW) culture medium (APW: 1% tryptone, 0.5% yeast extract, 1% NaCl, pH 8.2–8.3) at 37°C, while the other strains were cultured in Luria–Bertani (LB) culture medium (LB broth: 1% tryptone, 0.5% yeast extract, 1% NaCl, pH 7.4) at 37°C. Pure cultures with the growth of bacteria at the logarithmic phase, when living bacteria were far more numerous than dead ones, were collected though rinsing and re-suspension using sterilized saline, and then the bacterial concentrations were determined using plate counts.

BALB/c mice (8 weeks old) were immunized using *V*. *cholerae* O1 and O139 that were inactivated using a formaldehyde solution. The antibodies used for strip fabrication were selected for their high specificities and good coverage of widely diverse isolates using an enzyme-linked immunosorbent assay plates coated with bacterial strains that are closely genetically related to *V*. *cholerae*, other food-borne bacteria, or various *V*. *cholerae* O1 and O139 isolates from different sources. Finally, monoclonal antibodies against *V*. *cholerae* O1 (6G9 and 5F4) and *V*. *cholerae* O139 (M43H5) were chosen and purified using a Protein A column.

### Fabrication of the strips and detection

The UPT-LF strips for detection of *V*. *cholerae* serogroups O1 or O139 were named VchO1-UPT-LF or VchO139-UPT-LF, respectively, while the strip for simultaneous detection of *V*. *cholerae* serogroups O1 and O139 was named Vch-UPT-LF.

UCPs were functionalized with amino- and aldehyde-groups as previously described [31]. Functionalized UCPs (0.5 mg mL^−1^) were conjugated with antibodies (5F4, M43H5, or goat IgG) in Na_2_CO_3_-NaHCO_3_ buffer (50 mM, pH 9.5) through stirring for 1 h, and then BSA was used to block non-specific binding sites. After centrifugation, UCP-5F4, UCP-M43H5 and UCP-goat IgG conjugates were collected for further use.

For the VchO1-UPT-LF strip, 1.5 mg mL^−1^ 6G9 was dispensed onto the nitrocellulose membrane at a rate of 2 μL cm^−1^ to form a test line (T line), and 1.4 mg mL^−1^ UCP-5F4 conjugate was sprayed on the glass fiber to serve as the conjugate pad of the strip. For the VchO139-UPT-LF strip, 2.5 mg mL^−1^ M43H5 was dispensed as the T line, and 1.4 mg mL^−1^ UCP-M43H5 was used for the conjugate pad. For these two strips, goat anti-mouse IgG at 2 mg mL^−1^ was dispensed onto the nitrocellulose membrane as the control line (C line).

For the Vch-UPT-LF strip, 1.5 mg mL^−1^ 6G9, 2.5 mg mL^−1^ M43H5, and 2 mg mL^−1^ of rabbit anti-goat IgG were dispensed onto the nitrocellulose membrane to form the T1 line (channel 1 for detecting *V*. *cholerae* O1), T2 line (channel 2 for detecting *V*. *cholerae* O139), and the C line, respectively, whereas 2 mg mL^−1^ UCP-5F4, 2 mg mL^−1^ UCP-M3H5, and 1 mg mL^−1^ UCP-goat IgG were mixed together in equal volumes and sprayed onto the glass fiber to serve as the conjugate pad.

Slivers of nitrocellulose membrane, conjugate pad, sample pad (glass fiber), and absorbent paper were stuck onto the lamination pad, and then cut into 4-mm-wide pieces to install in the plastic cartridges. The overall scheme used for operating the device was the same as that described previously [29]. Detection samples were diluted in sample-treating buffer (pH7.2 0.03 mol L^−1^ phosphate buffer containing 0.5 mol L^−1^ NaCl, 0.5% NP-40) at a ratio of 1:9, and then 140 μL of the mixture was applied to the UPT-LF strip. After 15 min, the strips were scanned using a UPT biosensor, and each signal peak on the membrane was assessed. The areas of the peaks corresponding to the test and control lines are referred to as T and C values, respectively, and the T/C ratio is taken as the final measurement. The areas of the two T lines in the dual-target assay can both be calculated by the biosensor by increasing the signal recognition channel in the software program, based on that of a single-target assay. The detection systems were optimized by changing the components in the sample-treating buffer.

### Sensitivity, linearity, precision, and specificity assessments

For sensitivity evaluations, *V*. *cholerae* O1 at a concentration of 10^4^–10^8^ colony-forming units (CFU) mL^−1^ was prepared in phosphate buffer (PB), and then applied to the VchO1-UPT-LF and Vch-UPT-LF strips with PB serving as a blank control. *V*. *cholerae* O139 at a concentration of 10^4^–10^8^ CFU mL^−1^ was applied to the VchO139-UPT-LF and Vch-UPT-LF strips. Each sample was tested by one strip type on three occasions. PB were tested 10 times as blank control, and the mean ± three standard deviations of the T/C ratios was set as a cutoff threshold. Samples with T/C ratio higher than the cutoff were identified as positive samples. The data for the measured T/C values and bacterial concentration were analyzed by plotting a scatter diagram with linear fitting to evaluate the accuracy of the quantitation results. The ratio of the standard deviation and mean of the T/C values for three repeat tests, namely, the coefficients of variation, were calculated to evaluate the precision of the assay.

For specificity evaluations, 15 non-O1/non-O139 and 15 O139 *V*. *cholerae* isolates were analyzed using the VchO1-UPT-LF strip, while 15 non-O1/non-O139 and 15 O1 *V*. *cholerae* isolates were analyzed using the VchO139-UPT-LF strip. In addition, seven *Vibrio* spp. strains that are closely genetically related to *V*. *cholerae*, including, *V*. *fluvialis*, *V*. *metschnikovii*, *V*. *mimicus*, *V*. *parahaemolyticus* (two strains), and *V*. *vulnificus* (two strains), as well as nine food-borne bacteria that share similar transmission routes with *V*. *cholerae*, including *A*. *hydrophila* (two strains), *E*. *coli* (two isolates), *Salmonella* (three isolates), and *S*. *flexneri* (two isolates), were analyzed using the VchO1-UPT-LF, VchO139-UPT-LF, and Vch-UPT-LF strips. The concentrations of the bacterial samples used in the specificity evaluations were all 10^9^ CFU mL^−1^.

### Simultaneous detection of Vch-UPT-LF

First, *V*. *cholerae* O1 and O139 samples at a concentration of 10^6^–10^8^ CFU mL^−1^ were prepared in PB, and then each concentration of O1 was serially diluted ten-fold using O139 at 10^4^, 10^5^, 10^6^ CFU mL^−1^ respectively. Therefore, 10^5^–10^7^ CFU mL^−1^ of O1 mixed with 10^4^–10^6^ CFU mL^−1^ of O139 were prepared. The different concentrations of O139 mixed with O1 were also prepared with similar methods. The mixed samples were analyzed using Vch-UPT-LF.

### The detection time under pre-incubation conditions

Different concentrations of *V*. *cholerae* O1 and O139 were prepared by collection of pure cultures and ten-fold diluted cultures, and bacterial numbers were determined using plate counts. Subsequently, 1×10^1^–1×10^4^ CFU of bacteria were inoculated into 15 mL of APW broth, and then the pure cultures were analyzed using the Vch-UPT-LF strip three times after 0, 3, 4, 5, 6, and 7 h of incubation. With the incubation time prolonged, the T/C ratios will increase via bacterial propagation. The time corresponding to a point at which the increased T/C ratio exceeds the cutoff value is defined as the detection time [32].

### Field detection

One hundred and two field water samples were collected, while six samples were sterilized at 15 psi for 30 min using a high-pressure steam method and were used as negative controls. All the water samples, at a volume of 450 mL, were mixed with 300 μL of 1% potassium tellurite, 300 μL of 1 mol L^−1^ NaOH, and 50 mL of 10× APW broth for selective culture at 37°C for 18 h. The cultures were then simultaneously analyzed using Vch-UPT-LF, a colloidal gold immuno-chromatographic assay, real-time fluorescent PCR and a culture method.

A mixture of 14 μL of the culture sample and 126 μL of the sample-treating buffer was directly applied to the Vch-UPT-LF. *V*. *cholerae* rapid test kits (China Food and Drug Administration No. 3400100) for qualitative detection of O1 or O139 using a colloidal gold method, were obtained from Zhengzhou wantai biotech Co., Ltd. (Zhengzhou, China), and 100 μL of the culture samples was directly applied to the strip and the detection results were obtained through observation using the naked eye.

A SYBR green I real-time PCR method was applied as previously described [24,33] using a CFX96 Realtime-system (Bio-Rad). Chromosomal DNA was extracted from bacterial cultures using the QIAamp DNA mini Kit (QIAGEN, Hilden, Germany). A fragment of 83 bp was amplified from the O1 *rfb* gene using the forward primers (5’-GCG TAA ATA TCT AAA CGA TTG CAT TG-3’) and the reverse primers (5’-AAA CTC AGT TTC GAA GCG ATC AA-3’), while a fragment of 76 bp was amplified from the O139 *rfb* gene using forward primers (5’-GCG GTG TAG CGG GTT TTA TTA G-3’) and the reverse primers (5’-TGC ATA ATA CTT TCG ACC ATG GA-3’).

For the culture method (Diagnostic criteria for cholera, WS 289–2008, national health industry standards issued by the Minister of Health of the People’s Republic of China), the bacterial culture was inoculated in gentamycin agar culture medium (1% tryptone, 0.3% beef extract, 0.5% NaCl, 1% sucrose, 0.3% sodium citrate, 500 IU gentamycin, 1.5% agar, 0.0005% potassium tellurite) for selective culture at 37°C for 18 h. Subsequently, suspected colonies were screened using a slider agglutination test with diagnostic polyvalent serum for *V*. *cholera* O1 and O139. Positive colonies were inoculated and cultured on an LB agar plate for 18 h for further validation using diagnostic monovalent serum for *V*. *cholera* O1 or O139. All the diagnostic sera were saved by the Epidemiology and Chinese Center for Disease Control and Prevention (Beijing, China).

## Results and discussion

### Establishment of the UPT-LF assay

First, UPT-LF strips for separate detection of *V*. *cholerae* O1 or O139, namely VchO1-UPT-LF and VchO139-UPT-LF, were established. Bacteria in the samples were captured by UCP-5F4 or UCP-M43H5 in the conjugate pad, and then the complexes were further captured by 6G9 or M43H5 antibodies in the analytical membrane. The remaining UCP-5F4 or UCP-M43H5 was captured by goat anti-mouse IgG at the C lines.

**Fig 1:**
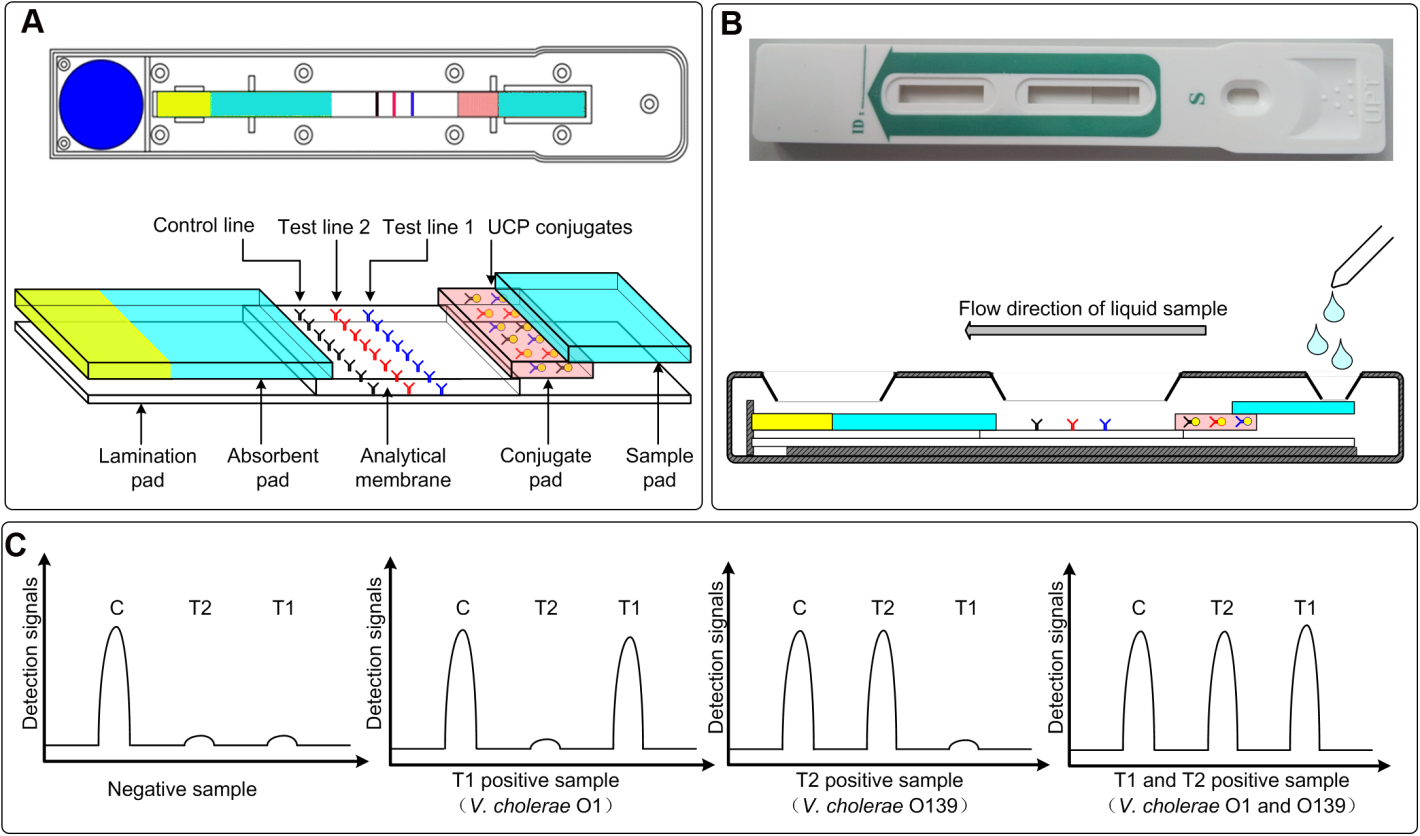
Schematic diagram for the Vch-UPT-LF assay. (A) An up-converting phosphor technology-based lateral flow assay for simultaneous detection of *V*. *cholerae* O1 and O139, namely Vch-UPT-LF, was established with antibodies against O1 and O139 as test line 1 and test line 2 dispensed on an analytical membrane at the strip, respectively. (B) After sample addition, the UCP-antibody complexes flowed forward with the liquid sample. (C) Positive signals were generated for test line 1 or test line 2 when the samples contained *V*. *cholerae* O1 or O139. The accuracy and precision of Vch-UPT-LF depended on stable signals on the C line, which were maintained by rabbit anti-goat IgG independently bound with UCP-goat IgG.

Subsequently, Vch-UPT-LF for simultaneous detection of *V*. *cholerae* O1 and O139 was established with 6G9 and M43H5 antibodies, and rabbit anti-goat IgG fabricated successively onto the analytical membrane as the T1 line, T2 line, and C line, respectively. Signals are generated at each line because of the capture of UCP-5F4, UCP-M43H5, and UCP-goat IgG conjugates when they flow forward with the added samples, either through direct capture or through a double antibody sandwich reaction (Fig 1). Finally, the T1/C and T2/C ratios calculated using the UPT biosensor were defined as the detection results for *V*. *cholerae* O1 and O139, namely channel 1 and 2, respectively.

Goat anti-mouse IgG was used as the C line of the VchO1-UPT-LF and VchO139-UPT-LF strips, leading to a decrease of signal on the C line with increasing concentrations of target bacteria. This intensifies the variation of T/C ratios and makes the detection more sensitive. However, signal fluctuation on the C line would make the detection inaccurate in terms of the choice of goat anti-mouse IgG used for the Vch-UPT-LF assay, because when a sample that is positive for only one of the two targets is detected, the decrease in signal on the C line caused by the single target would undoubtedly lead to an increase in the T/C ratio for the other target and generate false-positive results. Therefore, an independent reaction pair for the C line in the dual-target assay (i.e., rabbit anti-goat IgG and UCP-goat IgG) is used to increase the stability of the signals, thus ensuring the accurate detection of both *V*. *cholerae* O1 and O139.

### Sensitivity, linearity, and precision

A series of concentrations of *V*. *cholerae* O1 and O139 diluted using PB were analyzed using the VchO1-UPT-LF, VchO139-UPT-LF, and Vch-UPT-LF strips, with PB serving as a blank control. The sensitivity was defined as the lowest concentration at which the T/C ratios were higher than the cutoff threshold. The sensitivity of the VchO1-UPT-LF and VchO139-UPT-LF strips was 10^4^ CFU mL^−1^, whereas that of the Vch-UPT-LF strip for *V*. *cholerae* O1 and O139 was 10^5^ CFU mL^−1^. Despite the lower sensitivity, Vch-UPT-LF is applicable for field tests because the requirement of pre-incubation for detection on-site can increase the detection rates significantly, as described below.

**Fig 2:**
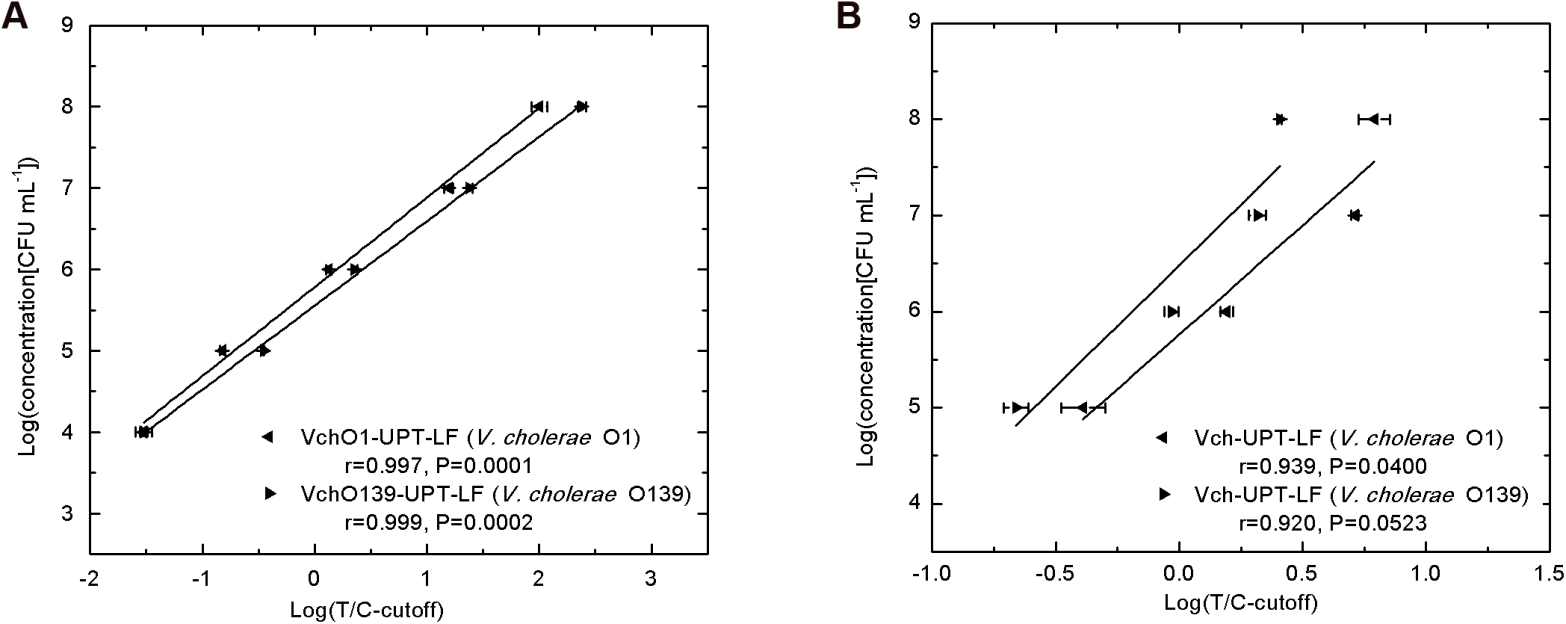
Standard quantification curves of the strips. (A) The sensitivity of the up-converting phosphor technology-based lateral flow (UPT-LF) assay for detection of *V*. *cholerae* O1 and O139, namely VchO1-UPT-LF and VchO139-UPT-LF, was 10^4^ CFU mL^−1^ with quantification ranges of 10^4^ to 10^8^ CFU mL^−1^. (B) *V*. *cholerae* O1 and O139 at concentrations from 10^5^ to 10^8^ CFU mL^−1^ could be detected quantitatively using the UPT-LF assay for simultaneous detection of O1 and O139 (Vch-UPT-LF). The coefficients of variation for all tests were less than 15%.

Standard quantification curves were generated by plotting the logarithm of the T/C-cutoff values versus the logarithm of the bacterial concentrations. The quantification ranges of VchO1-UPT-LF and VchO139-UPT-LF were both 10^4^ to 10^8^ CFU mL^−1^, while those of Vch-UPT-LF were 10^5^ to 10^8^ CFU mL^−1^ for both *V*. *cholerae* O1 and O139. The correlation coefficients of the linear regression analysis for standard quantification curves are all greater than 0.9, demonstrating that the quantitative results are highly accurate (Fig 2). The coefficients of variation are all less than 15%, demonstrating the precision of the quantification.

### Specificity

**Fig 3:**
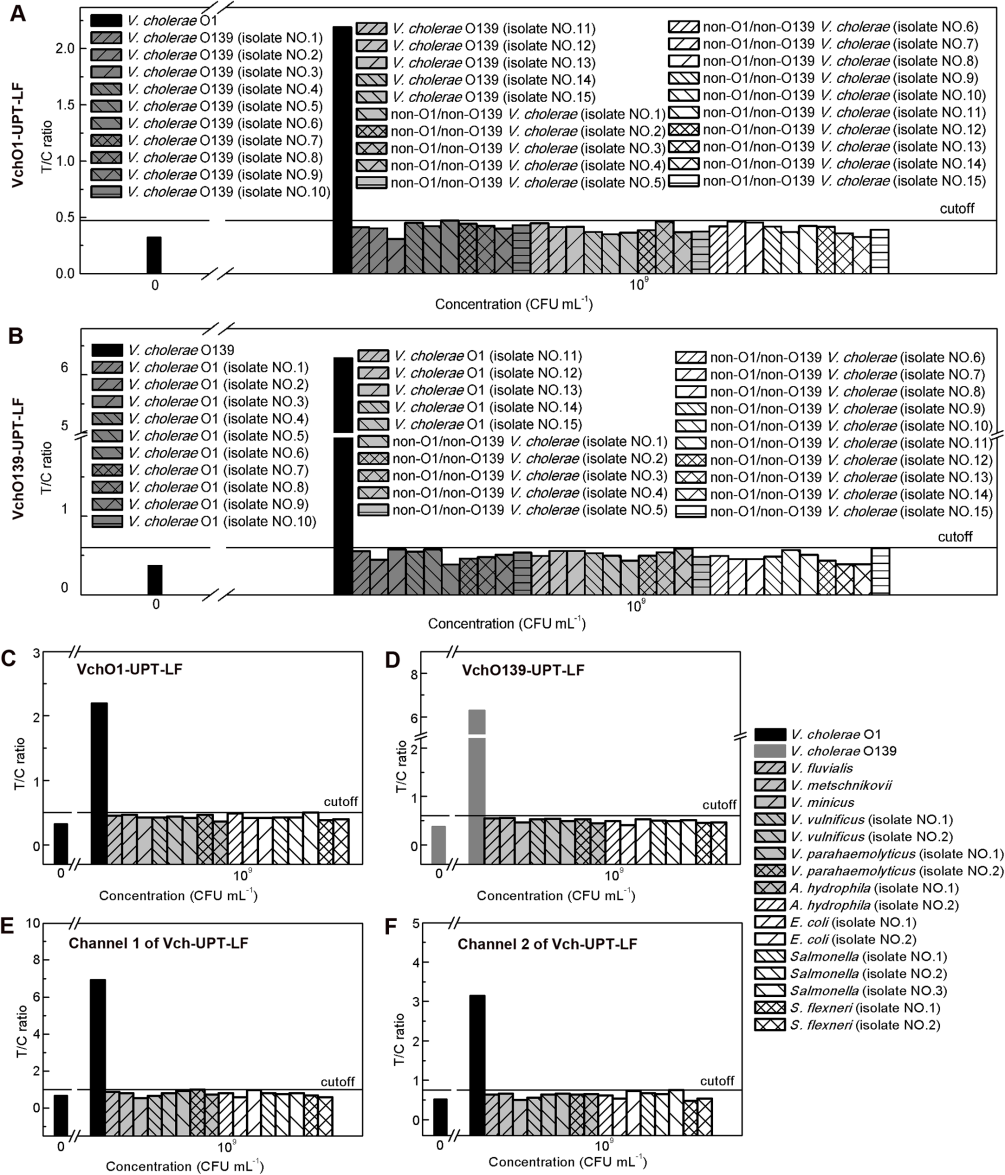
Specificity of the UPT-LF strips. (A) VchO1-UPT-LF and (B) VchO139-UPT-LF, namely the up-converting phosphor technology-based lateral flow (UPT-LF) assay for detection of O1 or O139, showed excellent specificity for 15 non-O1/nonO139 *V*. *cholerae* strains, and they are also specific for 15 O139 and 15 O1 isolates respectively. (C) VchO1-UPT-LF, (D) VchO139-UPT-LF, (E) channel 1 and (F) channel 2 of the UPT-LF strip for simultaneous detection of O1 and O139 (Vch-UPT-LF), did not generate false-positive results for seven other *vibrio* species and nine food-borne pathogens.

The specificity of each strip was evaluated using 10^9^ CFU mL^−1^ of bacteria that are closely genetically related to *V*. *cholerae* O1 and O139 or strains with similar transmission routes as *V*. *Cholerae*. VchO1-UPT-LF and VchO139-UPT-LF were specific to 15 isolates of *V*. *cholerae* O139 and O1, respectively, while they both showed excellent specificity for 15 non-O1/non-O139 *V*. *cholerae* isolates (Fig 3A and 3B). Furthermore, no false-positive results were generated by VchO1-UPT-LF (Fig 3C), VchO139-UPT-LF (Fig 3D) and Vch-UPT-LF (Fig 3E and 3F) for seven *Vibrio* species that are highly related genetically with *V*. *cholerae* and nine food-borne pathogens that can be transmitted by contaminated water or food like *V*. *cholerae*. These results demonstrate that the antibodies on the strips are very specific, providing a good foundation for simultaneous detection of *V*. *cholera* O1 and O139.

### Simultaneous detection of V. cholerae O1 and O139 using the Vch-UPT-LF strip

**Fig 4:**
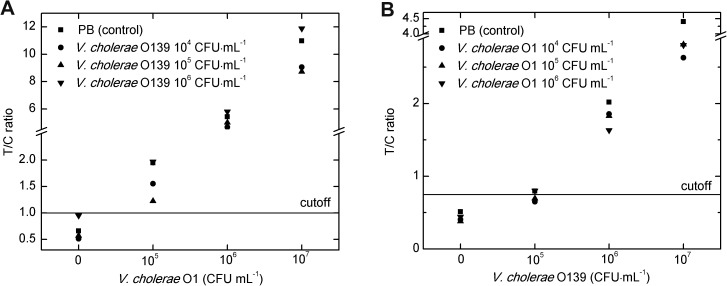
Simultaneous detection of *V*. *cholerae* O1 and O139 using the Vch-UPT-LF strip. (A) For simultaneous detection of O1 and O139 at a series of concentrations, the sensitivity of channel 1 of the up-converting phosphor technology-based lateral flow assay for simultaneous detection of O1 and O139 (Vch-UPT-LF) for detecting *V*. *cholerae* O1 was maintained. (B) The sensitivity of channel 2 of Vch-UPT-LF for detecting *V*. *cholerae* O139 decreased ten-fold. All quantification deviations were less than one order of magnitude.

Different concentrations of *V*. *cholerae* O1 or O139, mixed with different concentrations of O139 or O1, respectively, were detected by channel 1 or 2 of the Vch-UPT-LF strips. For channel 1, the T1/C ratios for *V*. *cholerae* O1 decreased slightly in the presence of low concentrations (10^4^ and 10^5^ CFU mL^−1^) of O139, whereas a high concentration (10^6^ CFU mL^−1^) of O139 slightly increased the T1/C ratios (Fig 4A). For channel 2, all of the T2/C ratios for *V*. *cholerae* O139 decreased slightly in the presence of *V*. *cholerae* O1, compared with the control group (Fig 4B).

As a whole, the sensitivity of the Vch-UPT-LF strip for the detection of *V*. *cholerae* O1 was unaffected by the presence of O139, while the sensitivity for O139 decreased ten-fold in the presence of O1. It is possible that the T2 line for detection of O139 was further away from the sample pad and can be influenced by the complex mass of O1 combined on the T1 line, meaning that T2/C ratio is much more susceptible to *V*. *cholerae* O1 in the samples. Fortunately, under the condition of interference of O139 or O1 to channels 1 or 2, the quantification deviations were all less than one order of magnitude for all simultaneous tests when compared with the control (Fig 4).

### Detection time of Vch-UPT-LF under pre-incubation conditions

**Fig 5:**
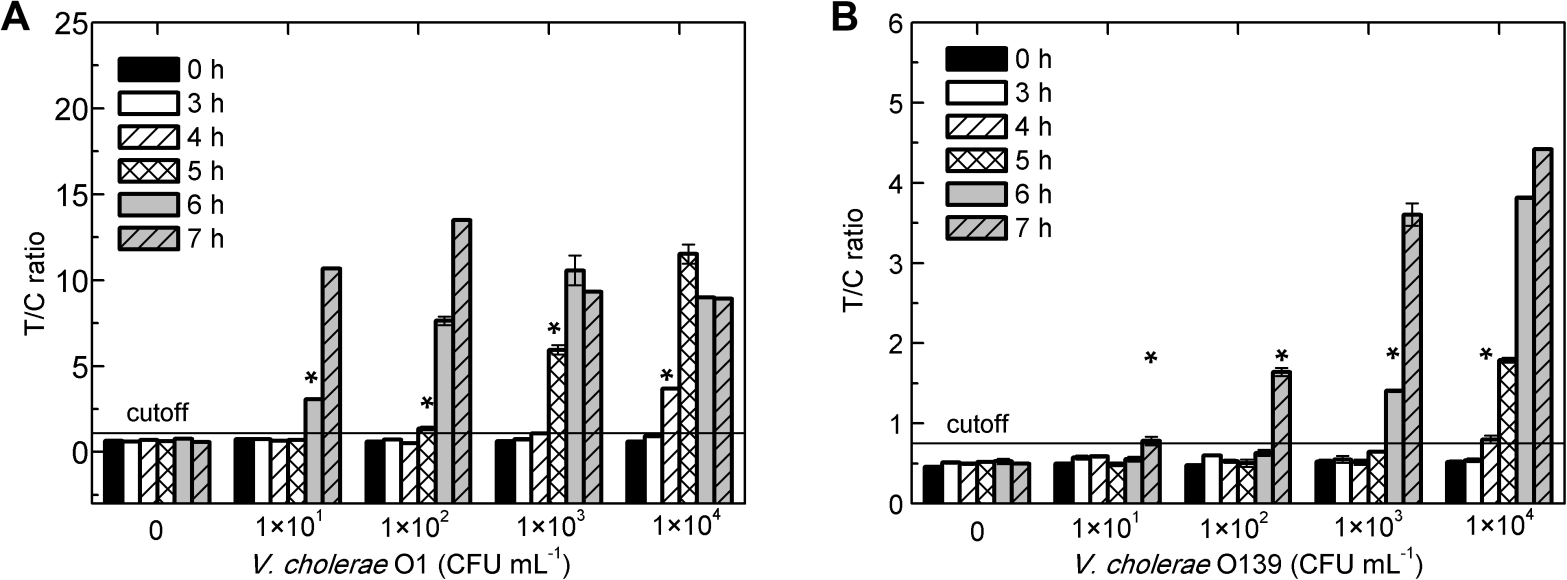
The detection times of the Vch-UPT-LF assay under pre-incubation conditions. The shortest incubation time, when the T/C ratios of the up-converting phosphor technology-based lateral flow assay for simultaneous detection of O1 and O139 (Vch-UPT-LF) were higher than the cutoff value, was defined as the detection time (*). (A) The detection times of channel 1 of Vch-UPT-LF for detecting *V*. *cholerea* O1. (B) The detection times of channel 2 of Vch-UPT-LF for detecting *V*. *cholerae* O139. The detection times were all less than 7 h for 10^1^ ~ 10^4^ CFU mL^−1^ of *V*. *cholerae* O1 and O139, while they could reach 4 h for both O1 and O139 at 10^4^ CFU mL^−1^.

*V*. *cholerae* strains are not permitted in foods according to the regulations of many international organizations, and low detection limits have promoted the extensive application of pre-incubation to improve detection rates. The time cost for pre-incubation, which must be determined for practical application of the method, hinges on the initial cell concentration of the sample and detection limit of the assay. Therefore, the shortest pre-incubation time for which the increased T/C ratio was higher than the cutoff value, namely the detection time [32], was determined. After several hours of incubation of *V*. *cholerae* O1 and O139 at low concentrations, the cultures were detected using the Vch-UPT-LF strip. As expected [32], the detection time is shortened by an increase in the initial cell concentration (Fig 5). The detection times for *V*. *cholerae* O1 and O139 at 1×10^1^ CFU mL^−1^ were both less than 7 h, and they could be detected within 4 h at 1×10^4^ CFU mL^−1^ (Fig 5). That is, detection rates using the Vch-UPT-LF were significantly increased under pre-incubation conditions, and it can return results for samples with few bacteria after 7 h of pre-incubation.

### Field evaluation of water samples

One hundred and two water samples, including six negative controls, were analyzed using Vch-UPT-LF, a colloidal gold immuno-chromatographic assay (Wantai, China), real-time fluorescent PCR [24,33] and a culture method. For the 96 analyzed samples, consistent results were obtained for 77 samples using the four methods, including 69 negative samples and eight samples that were positive for *V*. *cholerae* O1.

The other 19 samples that yielded inconsistent results are listed in S1 Table. They were all confirmed as negative samples using the culture method. Although the culture method is the gold standard, it is acknowledged that its sensitivity is often too low. Therefore, the sample was considered positive if two or more of the other three methods yielded the same positive result when the culture method failed. Based on this criterion, the results for these 19 samples were determined as follows. (1) Fourteen were determined as negative samples because three of four methods produced the same results, including the culture method. False-positive results were generated for Vch-UPT-LF, real-time fluorescent PCR and colloidal gold strip for one (for *V*. *cholerae* O139), six (five for O1 and one for O139) and seven (five for O1, one for O139, and one for both O1 and O139) samples, respectively. (2) Three were determined as positive samples because they were positive for O1, as confirmed using all tests except the culture method. (3) The remaining two samples, determined as O1 and O139 samples using Vch-UPT-LF and real-time fluorescent PCR assays (whereas the colloidal gold yielded negative results or false-positive results for O1), were determined as positive samples. Then, the sensitivity, specificity, false-positive rate, and false-negative rate for the four methods were calculated. When pre-incubation was conducted, the sensitivity of the Vch-UPT-LF assay was equivalent with that of the real-time fluorescent PCR (which is higher than that of the culture method and the colloidal gold assay) with a lower false positive rate (Table 2). It is possible that the stability of the UCPs, as well as the covalent bond between the UCPs and the antibodies, makes the Vch-UPT-LF assay more tolerant to interfering compounds in the water samples [28,29].

**Table 2:** The sensitivity and specificity of the four methods when analyzing field samples.

Detection target	Method	Sensitivity	Specificity	False-positive rate	False-negative rate
**O1**	**Culture**	66.67%	100%	0	33.33%
	**Vch-UPT-LF**^**a**^	100%	100%	0	0
	**Real-time fluorescent PCR**	100%	94.05%	5.95%	0
	**Colloidal gold assay**	91.67%	91.67%	8.33%	8.33%
**O139**	**Culture**	0^b^	100%	0	100%^b^
	**Vch-UPT-LF**	100%	98.95%	1.05%	0
	**Real-time fluorescent PCR**	100%	98.95%	1.05%	0
	**Colloidal gold assay**	0^b^	97.89%	2.11%	100%^b^

^a^ the up-converting phosphor technology-based lateral flow assay for the rapid and simultaneous detection of *V*. *cholerae* serogroups O1 and O139

^b^ The low sensitivity (0) and the high-false negative rate (100%) for *V*. *cholerae* O139 for the culture method and colloidal gold assay occurred because there was only one positive sample for *V*. *cholerae* O139, while both of these methods yielded false-negative results.

## Conclusions

An UPT-LF assay, Vch-UPT-LF, for the rapid, simultaneous detection of *V*. *cholerae* O1 and O139 was developed and evaluated as a POCT method in this study. Quantification detection of two targets in one strip by immobilizing two specific detection antibodies in adjacent positions of the nitrocellulose membrane was achieved for the first time, a feature that is distinct from single-target quantitation detection [28,29], multiplexed qualitative detection [26], and multiplexed quantitation detection via simple assemblage of many single-target strips in a disc [30]. Without the intensified signals from the contribution of the dependent C line as that of the single-target assay (VchO1-UPT-LF and VchO139-UPT-LF), the sensitivity of the Vch-UPT-LF assay’s two channels for detecting *V*. *cholerae* O1 and O139 decreased from 10^4^ to 10^5^ CFU mL^−1^, while fortunately, the quantification ranges still covered four orders of magnitude. The specificities of Vch-UPT-LF assay were as excellent as that of single-target assays for other bacterial strains that are closely genetically related to, or have similar transmission routes as, *V*. *cholerae*. The quantification results fluctuated as a result of the interaction effects between the two adjacent T lines, but the deviations were less than one order of magnitude in the presence of both O1 and O139. After 7 h of pre-incubation, 1×10^1^ CFU mL^−1^ of O1 and O139 could be detected. Field evaluation of water samples demonstrated that Vch-UPT-LF is as sensitive as real-time fluorescent PCR with a lower false positive rate under pre-incubation conditions, implying it is a candidate POCT method for use in a surveillance system for the prevention and control of epidemic cholera.

## Supporting information

S1 Table The 19 samples with inconsistent results from the four methods that were used for field detection. (DOCX)
