# Supplementary material for: Development and evaluation of an up-converting phosphor technology-based lateral flow assay for the rapid, simultaneous detection of Vibrio cholerae serogroups O1 and O139
Source: PLoS One. 2017 Jun 29;12(6):e0179937. doi: 10.1371/journal.pone.0179937 (PMC5491072; doi:10.1371/journal.pone.0179937)
Supplement: S1 Table — (DOCX) [file pone.0179937.s001.docx]

**S1 Table. The 19 samples with inconsistent results from the four methods that were used for field detection.**

| **Category** | **Sample No.** | **Judging criteria** | | **Culture method** | | **Vch-UPT-LF** | | **Real-time fluorescent PCR** | | **Colloidal gold assay** | |
| --- | --- | --- | --- | --- | --- | --- | --- | --- | --- | --- | --- |
|  |  | **O1** | **O139** | **O1** | **O139** | **O1** | **O139** | **O1** | **O139** | **O1** | **O139** |
| **Negative samples** | **610** | － | － | － | － | － | ＋ | － | － | － | － |
|  | **173** | － | － | － | － | － | － | ＋ | － | － | － |
|  | **179** | － | － | － | － | － | － | ＋ | － | － | － |
|  | **581** | － | － | － | － | － | － | ＋ | － | － | － |
|  | **582** | － | － | － | － | － | － | ＋ | － | － | － |
|  | **586** | － | － | － | － | － | － | ＋ | － | － | － |
|  | **154** | － | － | － | － | － | － | － | ＋ | － | － |
|  | **180** | － | － | － | － | － | － | － | － | ＋ | － |
|  | **185** | － | － | － | － | － | － | － | － | ＋ | － |
|  | **189** | － | － | － | － | － | － | － | － | ＋ | － |
|  | **595** | － | － | － | － | － | － | － | － | ＋ | － |
|  | **604** | － | － | － | － | － | － | － | － | ＋ | － |
|  | **167** | － | － | － | － | － | － | － | － | － | ＋ |
|  | **589** | － | － | － | － | － | － | － | － | ＋ | ＋ |
| **Positive samples** | **149** | ＋ | － | － | － | ＋ | － | ＋ | － | ＋ | － |
|  | **585** | ＋ | － | － | － | ＋ | － | ＋ | － | ＋ | － |
|  | **599** | ＋ | － | － | － | ＋ | － | ＋ | － | ＋ | － |
|  | **174** | ＋ | － | － | － | ＋ | － | ＋ | － | － | － |
|  | **590** | － | ＋ | － | － | － | ＋ | － | ＋ | ＋ | － |
